# Atypical Familial Amyotrophic Lateral Sclerosis Secondary to Superoxide Dismutase 1 Gene Mutation With Coexistent Axonal Polyneuropathy: A Challenging Diagnosis

**DOI:** 10.7759/cureus.20989

**Published:** 2022-01-06

**Authors:** Seraj Makkawi, Abdulaziz A Alqarni, Himyan Alghaythee, Suzan Y Alharbi, Anmar Fatani, Reem Adas, Ahmad R Abuzinadah

**Affiliations:** 1 College of Medicine, King Saud Bin Abdulaziz University for Health Sciences, Jeddah, SAU; 2 Research and Development, King Abdullah International Medical Research Center, Jeddah, SAU; 3 Department of Medicine, Ministry of the National Guard-Health Affairs, Jeddah, SAU; 4 Ophthalmology, Jeddah Eye Hospital, Jeddah, SAU; 5 Department of Medical Imaging, Ministry of the National Guard-Health Affairs, Jeddah, SAU; 6 Faculty of Medicine, King Abdulaziz University, Jeddah, SAU; 7 Neurology Division-Internal Medicine Department, King Abdulaziz University Hospital, Jeddah, SAU

**Keywords:** saudi arabia, polyneuropathy, sod1 gene mutation, familial amyotrophic lateral sclerosis, amyotrophic lateral sclerosis

## Abstract

Amyotrophic lateral sclerosis (ALS), also known as Lou Gehrig's disease, is a neurodegenerative disease that involves both the upper and lower motor neurons. Familial ALS, including superoxide dismutase 1 (SOD1) mutation, accounts for 5-10% of all cases of ALS. Typically, the symptoms of ALS are purely motor, though coexistent sensory symptoms have been reported in rare cases. In this report, we describe the case of a 47-year-old man who presented with progressive bilateral lower limb weakness and numbness for the last four years. A nerve conduction study (NCS) showed evidence of coexistent axonal sensorimotor polyneuropathy in addition to the typical findings of ALS in needle electromyography. Genetic testing confirmed the diagnosis of familial ALS secondary to the SOD1 genetic mutation. This report highlights that the presence of sensory symptoms should not exclude the possibility of ALS in an appropriate clinical setting.

## Introduction

Amyotrophic lateral sclerosis (ALS), often called Lou Gehrig's disease, is a neurodegenerative disease that causes muscle weakness and subsequent paralysis due to the involvement of both upper and lower motor neurons [1], eventually contributing to severe disability and death due to respiratory failure [2]. The incidence of ALS ranges from two to three cases per 100,000 people in Europe [1]. Male sex, white ethnicity, and age ≥60 years have been reported to be the major predisposing factors for ALS [3]. ALS is mainly classified into two types: sporadic, which accounts for 90-95% of cases, and familial, which accounts for 5-10%. The pathogenesis of ALS remains unknown; however, ribonucleic acid processing and protein clearance defects play a major role in the development of ALS. Familial ALS is most commonly caused by repeat expansions in the chromosome 9 open reading frame 72 gene followed by mutations in the superoxide dismutase 1 (SOD1) gene [4]. It is mainly diagnosed based on clinical features supported by electrophysiology, laboratory testing, and/or genetic testing after excluding other potential mimicking entities. Generally, it is believed that the symptoms of ALS are purely motor. However, recent evidence suggests a sensory nervous system involvement in certain cases with abnormal sensory nerve conduction study (NCS) findings, which has given rise to the question of whether or not ALS patients with sensory abnormalities should be classified under a new subtype of ALS [5,6]. We report a case of atypical familial ALS secondary to SOD1 mutation with coexistent axonal polyneuropathy.

## Case presentation

This case involves a 47-year-old man who was referred to our center for evaluation of possible polyneuropathy. His symptoms had started four years prior to the presentation as bilateral leg weakness, affecting the right leg more than the left, and had been associated with bilateral foot numbness. These symptoms had been gradual and progressive. He had then undergone a sleeve gastrectomy three years ago for obesity [body mass index (BMI)=40.8 kg/m^2^], hoping to improve his symptoms by reducing his weight. However, the weakness had continued to progress and resulted in difficulty in walking after surgery. Over the past year, his speech had slurred, and he had noted the development of slight hand weakness. Furthermore, he had urinary and bowel incontinence, in addition to difficulty with balance. However, he had no swallowing difficulty, diplopia, or other neurological symptoms. His systemic review was unremarkable. He had a medical history of hypertension. He was a non-smoker, and his family history was significant for an undiagnosed progressive muscle weakness involving his brother, whose symptoms had begun at 27 years of age and who had eventually died five years after due to respiratory failure.

On examination, the patient had slurred speech and mild bilateral facial weakness. The rest of the cranial nerve examinations were normal. Motor examination of the upper limbs revealed a mild spastic catch in the left arm. He also had significant wasting in the abductor pollicis brevis and first dorsal interosseous muscles bilaterally. The muscle power grades according to the Medical Research Council (MRC) grading were as follows: deltoid and biceps: 5/5 bilaterally; triceps: 4/5 bilaterally; wrist extension: 5/5 on the right and 4/5 on the left. The abductor pollicis brevis, first dorsal interosseous, and abductor digiti minimi had muscle grades of 1/5 bilaterally. Deep tendon reflexes were brisk in all limbs. Hoffman’s test results were positive bilaterally. Motor examination of the lower limb showed evidence of hypotonia, with minimal movement throughout (MRC grade 0-1 in all muscle groups), and Babinski sign bilaterally. Sensory examination revealed reduced pinprick in the lower limbs up to the ankles bilaterally, with reduced vibration at the toes.

Motor NCS (Table 1) showed a moderately prolonged distal latency with a severely reduced amplitude and borderline conduction velocity in the right median nerve motor potential. Moreover, a mildly prolonged distal latency with severely reduced amplitude and mildly reduced conduction velocity were noted in the right ulnar nerve motor potential. In addition, there was an absent response in the right tibial nerve at adductor hallucis and peroneal nerve at the extensor digitorum brevis. The right peroneal nerve at the tibialis anterior showed a reduced amplitude and a normal distal latency and conduction velocity. Sensory conduction studies (Table 2) of the right median and ulnar nerves showed mildly prolonged peak latencies with normal amplitude and mildly reduced conduction velocities. The right radial nerve was unremarkable. However, there was no response in the right sural nerve. Electromyography studies of the upper and lower limbs showed evidence of fibrillation, positive sharp waves, and rare fasciculations in the right abductor digiti minimi, first dorsal interosseous, tibialis anterior, medial gastrocnemius, and vastus lateralis. The right biceps, trapezius, extensor indices, triceps, and deltoid were all normal. The right T6 paraspinal muscles showed rare fasciculations, but no fibrillation or positive sharp waves were noted. Hence, the overall evidence of widespread denervation involving the lumbar region and, to a lesser extent, the cervical region was noted.

**Table 1:** Motor nerve conduction study EDB: extensor digitorum brevis; TA: tibialis anterior; ms: millisecond; mV: millivolt; m/s: meters per second; NR: no response

Nerve	Site		Latency (ms)	Latency (normal value) [7]	Amplitude (mV)	Amplitude (normal value) [7]	Conduction velocity (m/s)	Conduction velocity (normal value) [7]
Median	Right	Wrist	7.4	≤4.4	1	≥4.0	-	-
		Elbow	12.1	-	0.8	-	48.9	≥49
Ulnar	Right	Wrist	5.6	≤3.3	1.7	≥6.0	-	-
		Elbow	10	-	1.2	-	47.7	≥49
		Above elbow	11.9	-	1.1	-	51.3	≥49
Peroneal (EDB)	Right	Ankle	NR	≤6.5	NR	≥2.0	NR	-
		Head of fibula		-		-		≥44
		Popliteal		-		-		≥44
Peroneal (TA)	Right	Head of fibula	3.6	≤6.7	1	≥3.0	-	-
		Popliteal	5.6	-	1	-	45	≥44
Tibial	Right	Ankle	NR	≤5.8	NR	≥4.0	NR	-
				-		-		≥41

**Table 2:** Sensory nerve conduction study ms: millisecond, µV: microvolts, m/s: meters per second, NR: no response

Nerve	Site	Peak latency (ms)	Peak latency (normal value) [7]	Amplitude (µV)	Amplitude (normal value) [7]	Conduction velocity (m/s)	Conduction velocity (normal value) [7]
Median	Right	4.8	≤3.5	34.8	≥20	41.9	≥49
Ulnar	Right	3.4	≤3.1	22.5	≥17	47.8	≥49
Radial	Right	2.9	≤2.9	23.3	≥15	49.8	≥49
Sural	Right	NR	≤4.4	NR	≥6	NR	≥40

**Figure 1:**
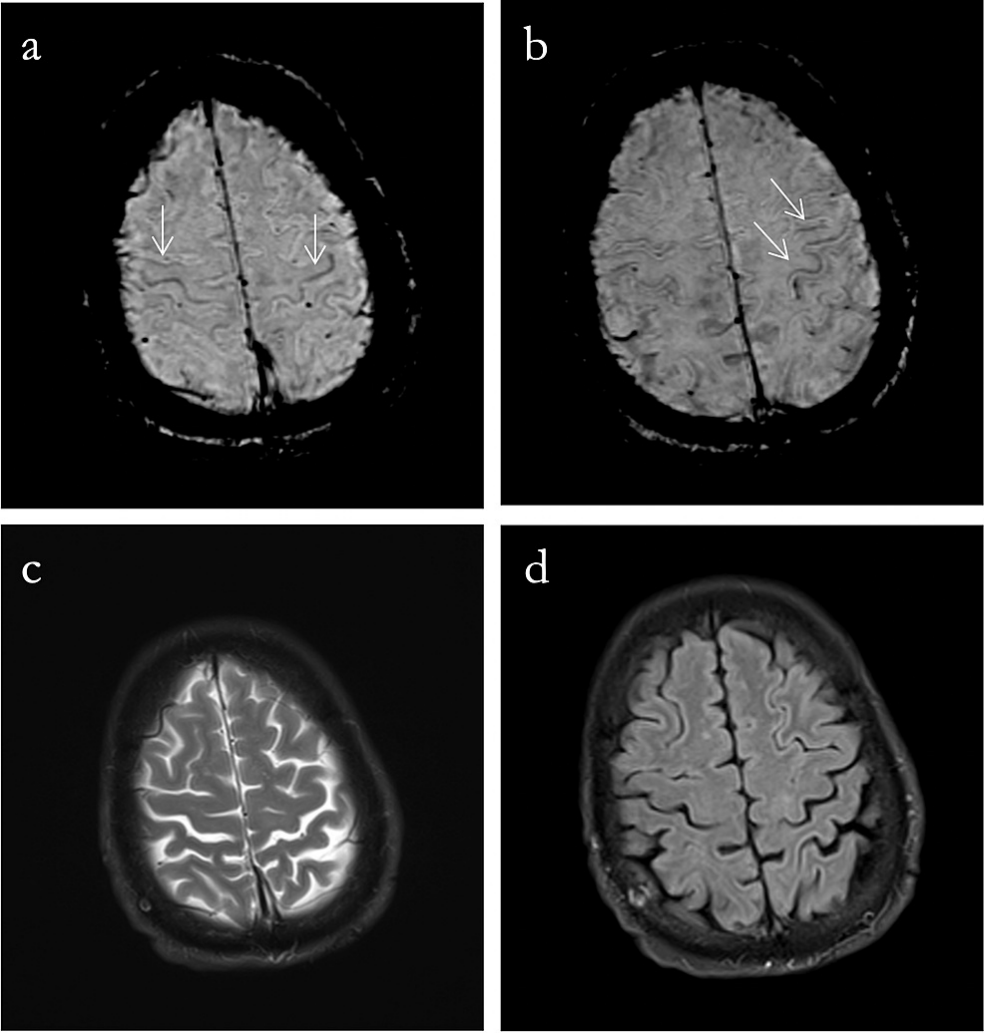
Brain MRI Axial susceptibility-weighted imaging images (a, b) show the classic “motor band sign,” which are curvilinear bands of low signal intensity solely observed in the primary motor cortex bilaterally (white arrows). Note that this finding is not seen on the corresponding axial T2 image (c). Axial fluid-attenuated inversion recovery image (d) shows subtle signal changes in the subcortical white matter, more on the left side, indicating the degeneration of the corticospinal tract MRI: magnetic resonance imaging

MRI of the brain and cervical spine (Figure 1) showed abnormal linear areas of blooming/iron deposition along the cortices of the precentral gyri, indicating a positive motor band sign. There was possible high signal intensity involving the ventral gray matter of the cervical cord extending from the C3-C6 spines. A mild volume loss was also noted in the cord. However, the image was degraded by motion artifacts.

Extensive workup, including routine basic screen, liver function, thyroid function, vitamin B12, vitamin E, copper, syphilis, HIV, Brucella, hepatitis B and C, QuantiFERON-TB Gold, anti-aquaporin 4 and anti-myelin oligodendrocyte glycoprotein (MOG) antibodies, vasculitis, and paraneoplastic screening, to rule out ALS mimickers was performed, and all yielded negative results. CT of the chest, abdomen, and pelvis was negative for lymphadenopathy or masses. On cerebrospinal fluid (CSF) analysis, the following were noted: protein level of 0.42 mg/dL; white blood cell count of 1; and negative bacterial culture and virology screening.

Furthermore, whole-exome sequencing testing was performed on the patient after obtaining informed consent. Sequence analysis identified a heterozygous variant, c.230A>T p. (Asp77Val), of the SOD1 mutation. The patient was counseled regarding the diagnosis and prognosis. He was referred to physiotherapy and occupational therapy for rehabilitation and to the swallowing and respiratory team for assessment. He was started on riluzole 50 mg twice per day.

## Discussion

Familial ALS accounts for only 5-10% of all ALS cases [4]. More than 20 genes are associated with familial or sporadic ALS, with the most common genetic causes being repeat expansions in the chromosome 9 open reading frame 72 gene, SOD1 mutations, and TAR DNA-binding protein 43 mutations [4,8]. SOD1, a metalloprotein consisting of 153 amino acids, is mainly found in the cytoplasm and, less commonly, in the nuclear compartment. The most common mutations in SOD1 are D90A, A4V, and G93A. Specifically, in the United States, the A4V mutation is the most common mutation among the population. These genetic mutations lead to the deposition of SOD1 misfolded protein aggregates in motor neurons, which induces toxicity [9]. The association between sensory axonal degeneration and the mutant SOD1 gene is supported by studies showing a link between the gene's dysfunction and sensory neuropathy [10]. Specifically, a study has revealed that the sensory action potentials and sural nerve conduction velocity decreased over six months in 50 patients with ALS, with 60% of patients exhibiting neurophysiological dysfunction in at least one afferent pathway [11].

It has been hypothesized that the first insult to the sensory system leads to dorsal root ganglia neuronopathy, followed by progressive sensory axonal atrophy, secondary demyelination, remyelination, and eventually axonal loss [12]. The majority of studied ALS models representing the overexpression of the mutated form of human SOD1 G93A involved transgenic mice and provided strong evidence for the dying-back pattern of degeneration. Transgenic mice expressing the human SOD1 G93A also displayed neurodegeneration in the sensory axons, dorsal root ganglion, and proprioceptive fibers of muscle spindles, reflecting the sensory dysfunction observed in human ALS. Damage to axons is detected as early as the pre-symptomatic stage, and it progresses in a distal-to-proximal gradient. In addition, the loss of dorsal root axons has also been observed in mice that were overexpressing the genetic mutants [13]. The magnitude of axonal pathology along with disease progression overrides the moderate loss of motor neurons in the spinal cord, suggesting that the motor phenotype in these mouse models is mostly directed by damage to the peripheral nervous system [14,15].

ALS typically manifests as adult-onset muscle weakness and wasting of the limb muscles, more commonly in the distal muscles. Bulbar involvement has been demonstrated in 25-30% of patients with ALS [9]. Bladder and bowel symptoms such as urinary urgency and frequency, constipation, and even incontinence have been occasionally associated with ALS [16]. Furthermore, 2-20% of patients with ALS complain of sensory problems, with numbness being the most common symptom, followed by neuropathic pain, tingling, and decreased temperature sensation [5]. Before the onset of fiber loss and axonal damage, an abnormality in the blood-nerve barrier has been demonstrated as ultrastructural evidence of ALS [17]. One study that combined spinal imaging and electrophysiological recordings in patients with an early diagnosis of ALS revealed sensory deficits and subclinical anatomical damage to the dorsal column [18]. However, sensory degeneration generally occurs to a lesser extent compared to motor degeneration [18]. These studies emphasize the previously raised question as to whether ALS patients with sensory abnormalities should be classified under a new type of ALS. These also further emphasize that ALS is clinically diagnosed, and abnormal sensory NCS findings on their own are not sufficient for ruling out ALS, as seen in our patient who had been misdiagnosed several times due to the coexistence of polyneuropathy [5,6].

The phenotype of our case also adds to the currently described features of familial ALS with SOD1 mutation. Such addition is important to help identify cases of SOD1 mutation as there is a potential therapeutic target. Tofersen is an antisense oligonucleotide that blocks translation and reduces SOD1 protein levels. It has been demonstrated that it reduces SOD1 concentration in the CSF, and that was associated with some efficacy in reducing the rate of deterioration in the functional and respiratory outcomes [19].

## Conclusions

ALS is primarily a motor neuron disease that progressively leads to severe disability and even death. Based on the available literature, the coexistence of sensory symptoms in ALS is rare, making its diagnosis challenging, and patients may remain misdiagnosed for years. Therefore, since ALS is diagnosed clinically, it is important to take a detailed history of the course and progression of symptoms and perform a thorough physical examination. Further studies should be utilized as an adjunct to the history and physical examination and not as a guide to the diagnosis. Thus, sensory symptoms and abnormal sensory NCS, as described in our case, should not exclude the possibility of ALS in an appropriate clinical setting.
